# Association between hand grip strength and stroke in China: a prospective cohort study

**DOI:** 10.18632/aging.202630

**Published:** 2021-03-03

**Authors:** Guihao Liu, Yunlian Xue, Sheng Wang, Yuhu Zhang, Qingshan Geng

**Affiliations:** 1 Guangdong Provincial People's Hospital, Guangdong Academy of Medical Sciences, Guangzhou, Guangdong, China

**Keywords:** hand grip strength, stroke, incidence

## Abstract

Objectives: The association between weak hand grip strength (HGS) and stroke recovery has been studied; however, few studies focused on the association of HGS with stroke prevalence and incidence.

Methods: A prospective cohort baseline study of a nationally representative sample in Chinese adults aged 45 years and older in 2011 was followed up in 2015. 8871 participants without stroke at baseline were followed. The associations of HGS and its changes with stroke prevalence and incidence were investigated using logistic regression models and Cox proportional hazards regression models.

Results: Association of HGS and stroke prevalence was significant. HGS weakness significantly increased risk of stroke incidence, with 89.3% higher risk when compared to normal HGS. During 35,263 person-years of follow-up, 112 stroke patients occurred. The four-year incidence rate ratio of stroke for participants with a HGS weakness was 2.15, compared to normal HGS participants. HGS changes in weakness/weakness from 2011 to 2015(D-HGS) and normal/weakness D-HGS had higher risks of stroke incidence when compared with those who had normal/normal D-HGS.

Conclusions: HGS weakness and decline of HGS were associated with stroke incidence for adults aged 45 years and older in China.

## INTRODUCTION

Over the past four decades, stroke incidence rates have decreased by 42% in high-income countries and increased by >100% in low- and middle-income countries [1]. Stroke is the second most common cause of mortality worldwide and the first leading cause of death and disability in China [2, 3]. The prevalence and incidence of stroke have risen faster in China than in other countries [4]. The adverse consequences of stroke on physical health are becoming ever more serious [5]. Therefore, primary prevention is particularly important because >76% of strokes are first events [6].

According to the Sixth National Census of Population (6th NCP) in 2010 in China, the proportion of those aged 60years and older accounted for 13.26% of the population, representing a 2.93% increase over the prior 10years [7]. The risk of stroke rapidly rises with age, doubling each decade after age 55 [8]. Population aging has driven up the disease burden and, as a result, strokes have become the leading disease burden in China [9].

Hand grip strength (HGS) is a reliable measurement of localized muscle strength and correlates with physical activity [10], and can be recommended as a stand-alone measurement or as a component of a small battery of measurements for identifying older adults at risk of poor health status [11]. HGS has proven to be an important biomarker of health with high reliability and validity, which can be highly representative of a person’s current and prognostic health profile [12]. HGS weakness has proven to be linked to a range of health outcomes, including higher all-cause mortality rates [13], morbidity [14], recognition decline, and frailty [15]. HGS is a major diagnostic component of sarcopenia, which described as a general loss of muscle mass that occurred with advancing age, and has the same risk factor with stroke, such as age [14].

HGS is a useful biomarker to measure the recovery and prognostic after stroke [16], with decreased HGS forecasting worse survival and recovery after stroke [17]. The absence of measurable grip by one month after stroke indicates that there will be poor functional outcome [18]. HGS also significantly associated with cognitive function of stroke patients [19].

Grip strength measurement is appealing as a quick and inexpensive way to stratify an individual’s risk of stroke recovery. Although the association of HGS and prognosis of stroke was studied, little knowledge of HGS and incidence of stroke was known. A prospective study in 2015 revealed that every 5kg decline in grip strength was associated with 9% increase in the risk of having a stroke [20], which was an inspirational finding. However, the evidence was got from 17 countries. To our knowledge there have been no such studies in China. Therefore, understanding the relationship of HGS and stroke incidence would be useful as an inexpensive and simple risk-stratifying method for stroke prevention. So, we investigated the association of HGS with prevalence and incidence of stroke in a prospective national cohort in China. We also analyzed the association of changes in HGS in four years with incidence of stroke, which has never been reported before.

## RESULTS

A total of 12,237 participants were included in this baseline study. The average age of all participants was 59.01±9.60 years (range equals 45-95 years). Of the participants, 5,887(48.1%) were male and 6,350 (51.9%) were female. Of the participants, 11.6% (n=1415) demonstrated HGS weakness, 2.0% (n=245) had a stroke.

Table 1 demonstrates the association between covariates and grip strength at baseline. Participants with HGS weakness were more likely to be of an older age, not married, have an education level of below primary school, live in village, drink less than once a month or never drink, have lower BMI, higher glucose, lower total cholesterol, and a higher proportion of unhealthy hypertension, when compared with those whose HGS were normal.

**Table 1:** Baseline characteristics in two HGS groups.

	**Normal HGS (n=10822)**	**Weakness HGS (n=1415)**	**p value**
Age^a^, yrs	57.91(8.95)	67.4(10.24)	<0.001
Gender^b^, %			
Male	5178(47.85)	709(50.11)	0.110
Female	5644(52.15)	706(49.89)	
Marriage status^b^, %			
Married	9595(88.66)	1035(73.14)	<0.001
Others	1227(11.34)	380(26.86)	
Education^b^, %			
Below primary school	4678(43.23)	958(67.7)	<0.001
Primary school	2493(23.04)	273(19.29)	
Junior middle school	2362(21.83)	121(8.55)	
High shool	1076(9.94)	50(3.53)	
Junior college and above	211(1.95)	13(0.92)	
Place of residence^b^, %			
Village	8658(80.00)	1201(84.88)	<0.001
City or town	2097(19.38)	206(14.56)	
Others	67(0.62)	8(0.57)	
Smoking behavior^b^, %			
Not smoking	7178(66.46)	917(64.99)	0.273
Smoking	3623(33.54)	494(35.01)	
Alcoholic intake^b^, %			
Drink more than once a month	2780(25.69)	270(19.11)	<0.001
Drink less than once a month	880(8.13)	105(7.43)	
Never had a drink	7162(66.18)	1038(73.46)	
BMI ^a^, kg/m^2^	23.62(3.89)	22.22(4.39)	<0.001
Glucose ^c^, mg/dl	102.42(94.5-113.76)	103.14(95.22-116.46)	<0.001
Total Cholesterol ^c^, mg/dl	190.98(168.17-216.30)	185.37(161.21-209.54)	<0.001
CRP ^c^, mg/l	1.02(0.55-2.12)	1.26(0.62-2.99)	<0.001
Hypertension^b^, %			
Health	3554(33.12)	422(30.27)	0.033
Not health	7178(66.88)	972(69.73)	
Stroke			
Yes	191(1.76)	54(3.82)	<0.001
No	10631(98.24)	1361(96.18)	

Abbreviations: HGS, hand grip strength; BMI, body mass index, CPR, C-Reactive Protein. ^a^ *P* value based on two independent t tests. ^b^ *P* value based on χ^2^ test. ^c^ *P* value based on Wilcoxon W test.

Stroke prevalence rate in participants with HGS weakness was 3.8% (n=54) and in participants with normal HGS was 1.8% (n=191), which was significantly different (χ^2^=26.838, P<0.001) (Table 1). After following up four years in those without stroke at baseline, the stroke incidence rate in participants with HGS weakness was 2.4% (n=21) and in participants with normal HGS was 1.1% (n=91), which was significantly different (χ^2^=10.365, P=0.001).

Of all the 8,871 participants who were followed up in 2015, 9.8% (n=867) had baseline HGS weakness. During 35,263 person-years of follow-up (average follow up time of 3.98 years, median follow up time of 4 years), 1.3% (n=112) had a stroke. Four years incidence density (ID) of stroke was 614 per 100 thousand (incidence rate ratio (IRR)=2.15) for participants with HGS weakness, and 286 per 100 thousand (IRR=1) for participants with a normal HGS.

Of those 7,371 participants that had measured HGS in 2015, the results were as follows: 77.1% (n=5686) had normal/normal D-HGS, of which 0.8% (n=44) had a stroke; 4.6% (n=339) had weakness/normal D-HGS, of which 0.9% (n=3) had a stroke; 5.1% (n=375) had weakness/weakness D-HGS, of which 2.9% (n=11) had a stroke; 13.2% (n=971) had normal/weakness D-HGS, of which 2.4% (n=23) had a stroke (Table 2). Stroke incidence in four groups were significantly different (χ^2^=31.688, P<0.001). The 4-year incidence density (ID) of stroke was 222, 746, 599 and 194 per 100 thousand (IRR=1.14, 3.84, 3.09 and 1) for participants with a weakness/normal, weakness/weakness, normal/weakness and normal/normal D-HGS.

**Table 2:** Stroke incidence of HGS changing in 2011 to 2015.

	**N (%)**	**Stroke**	**No stroke**
normal/normal D-HGS	5686(77.14)	44(0.77)	5642(99.23)
weakness/normal D-HGS	339(4.6)	3(0.88)	336(99.12)
weakness/weakness D-HGS	375(5.09)	11(2.93)	364(97.07)
normal/weakness D-HGS	971(13.17)	23(2.37)	948(97.63)

**Figure 1:**
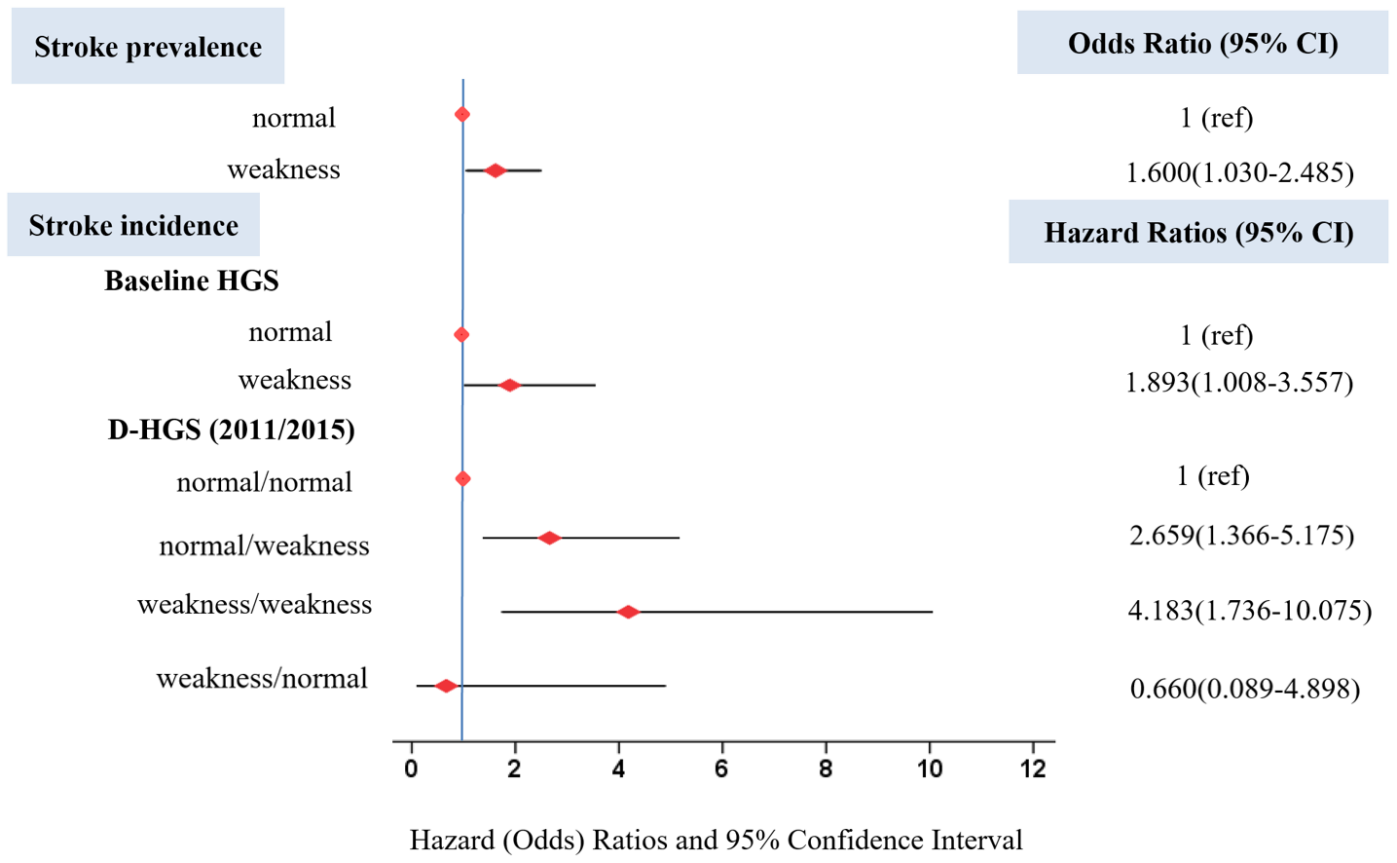
**Odds ratio for stroke prevalence and hazard ratios for stroke incidence based on HGS.** Hazard (Odds) ratios were adjusted for age, gender, marriage status, education level and place of residence, smoking behavior, alcoholic intake, BMI, hypertension, glucose, total cholesterol and C-reactive protein. Horizontal lines represent 95% confidence intervals. CI=confidence interval.

**Figure 2:**
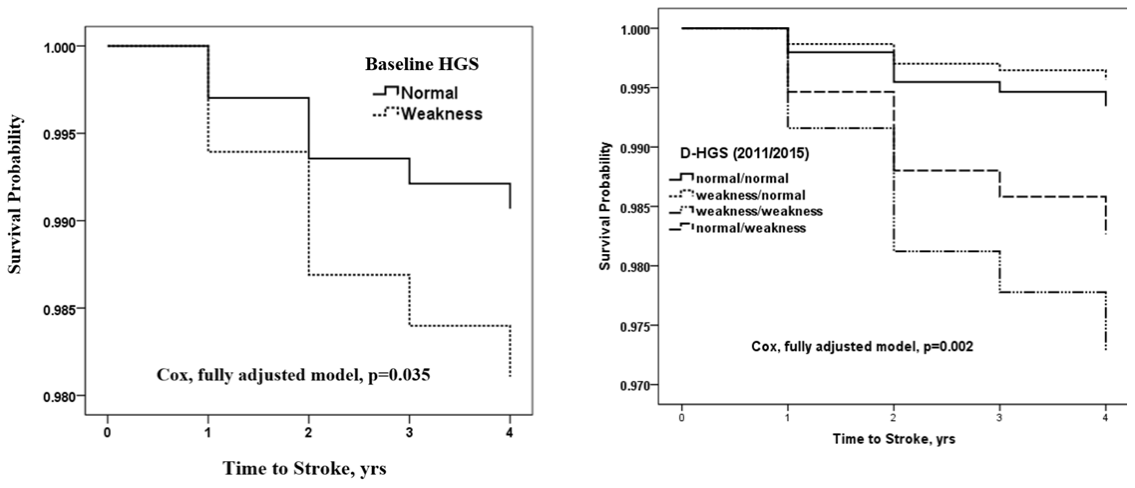
**Cox regression of fully adjusted model for the cumulative risk of Stroke grouped by HGS.** Fully adjusted model shows adjustments for age, gender, marriage status, education level and place of residence, smoking behavior, alcoholic intake, BMI, hypertension, glucose, total cholesterol and C-reactive protein. Abbreviations: 1. HGS: hand grip strength. 2. D-HGS: according to HGS status in 2011 and 2015, we classified all participants into four groups: normal/normal, weakness/normal, weakness/weakness and normal/weakness.

HGS had a higher stroke prevalence rate (odds ratio [OR]: 1.600; 95% confidence interval [CI]: 1.030 to 2.485) in the fully adjusted model (Table 3, Figure 1). For followed up participants, compared with those who had normal HGS, participants with HGS weakness had a higher risk of stroke incidence (hazard ratio [HR]: 1.893; 95% CI: 1.008 to 3.557) in fully adjusted model (Table 3, Figure 1). When considering the changes of HGS in four years, we found that compared to those who had normal/normal D-HGS, participants with weakness/weakness D-HGS and normal/weakness D-HGS had higher risks of stroke incidence (HR: 4.183; 95% CI: 1.736 to 10.075 and HR: 2.659; 95% CI: 1.366 to 5.175 respectively), but without significant association for weakness/normal D-HGS (HR: 0.660; 95% CI: 0.089 to 4.898) in the fully adjusted model. The cumulative risk of stroke for adults with baseline HGS weakness was significantly higher than with normal baseline HGS; for adults with weakness/weakness D-HGS was highest, following with normal/weakness D-HGS (Figure 2).

**Table 3:** Associations between HGS and stroke prevalence and incidence.

	**Unadjusted**	**Model 1**	**Model 2**	**Model 3**
Stroke Prevalence				
normal	1 (ref)	1 (ref)	1 (ref)	1 (ref)
weakness	2.208(1.624-3.003)	1.556(1.111-2.179)	1.526(1.089-2.137)	1.600(1.030-2.485)
Stroke Incidence				
Baseline HGS				
normal	1 (ref)	1 (ref)	1 (ref)	1 (ref)
weakness	2.142(1.333-3.442)	1.775(1.062-2.965)	1.796(1.073-3.004)	1.893(1.008-3.557)
D-HGS (2011/2015)				
normal/normal	1 (ref)	1 (ref)	1 (ref)	1 (ref)
weakness/normal	1.143(0.355-3.680)	1.233(0.378-4.025)	1.238(0.379-4.043)	0.660(0.089-4.898)
weakness/weakness	3.830(1.978-7.415)	3.669(1.759-7.653)	3.652(1.747-7.635)	4.183(1.736-10.075)
normal/weakness	3.082(1.861-5.103)	3.188(1.864-5.454)	3.053(1.771-5.265)	2.659(1.366-5.175)

Model 1: adjusted for age, gender, marriage status, education level and place of residence. Model 2 = Model 1+ smoking behavior, alcoholic intake. Model 3 = Model 2+ BMI, hypertension, glucose, total cholesterol and C-reactive protein.

Abbreviations: 1. BMI: body mass index; 2. HGS: hand grip strength; 3. D-HGS: according to HGS status in 2011 and 2015, we classified all participants into four groups: normal/normal, weakness/normal, weakness/weakness and normal/weakness.

## DISCUSSION

In this study, participants were from a nationally representative cohort in Chinese adults aged 45 years and older. We first analyzed the association of HGS and stroke prevalence in the baseline, and then followed up four years later with those participants without a stroke. We found that HGS weakness was significantly associated with higher stroke prevalence rate and incidence rate. The changes of HGS in four years were significantly associated with stroke incidence. The associations were independent of age, gender, marriage status, education level, place of residence, smoking behavior, alcoholic intake, BMI, hypertension, fasting plasma glucose, total cholesterol and CRP. HGS and HGS changes can be taken as effective biomarkers of stroke prevalence and incidence, which is meaningful due to the ease of measurement and assessment in clinical practice and, therefore, may be as a quick, low-cost screening tool to identify high-risk patients who may be susceptible to stroke.

In this study, we found that participants with HGS weakness had a 89.3% higher risk of stroke incidence. Compared with normal/normal D-HGS, participants with HGS weakness in 2011 and 2015 had a 318.3% higher risk of stroke incidence; participants with normal HGS in 2011 but HGS weakness in 2015 had a 165.9% higher risk of stroke incidence. Our findings are in line with previous studies. The HGS of the non-paretic hands in stroke patients was found to be obviously less than the HGS of the dominant hand in non-stroke controls, and was positively associated with functional improvement after rehabilitation [21]. HGS was indicated as a biomarker of recovery and prognostic of stroke [18]. Park indicated that decreasing of HGS had negative effects on functional recovery in subacute ambulatory stroke patients [17]. HGS was also found significantly associated with stroke mortality [19]. Taken together with previous studies, our findings underscore the importance of exercising HGS as a simple way to prevent stroke incidence.

An eight-year follow-up study showed that higher HGS was associated with lower levels of inflammation which may partly explained the association between HGS and mortality [22]. CRP is an inflammatory marker and positively associates with stroke [23]. We found participants with HGS weakness showed higher CRP level than those with normal HGS, which maybe a mechanism explaining the association weakness HGS with stroke prevalence and incidence. Second, we found that HGS weakness participants had higher levels of hypertension. As we know, hypertension is a risk factor for stroke, and had the greatest population attributable risk [24]. This maybe another possible reason for explaining the association of HGS and stroke. Thirdly, higher relative grip strength was significantly associated with lower glucose [25], which was the same with our study. A study investigated that incident stroke with hypertension and diabetes is quite high [26]. Furthermore, HGS is a simple measurement of physical capability [27]. HGS weakness relates to physical decline [28], which might bring high risk factors for stroke, such as lack of cardiopulmonary exercise [29]. However, in this study, we have adjusted for demographic factors including age, sex, marriage, education level and place of residence, biomarkers including BMI, glucose, total cholesterol and CRP, and lifestyle factors including alcoholic intake and smoking behavior, and the association between HGS and stroke prevalence and incidence remained significant.

### Strengths and limitations

The major strengths of this population-based study are; the use of a nationally representative sample, which facilitates generalization of the findings to the general population in China; the prospective cohort of the 4-year follow-up period; assessment of association of HGS changes during the follow-up with stroke; and the controlling of potential confounding effects from a variety of biomarkers, physiological index, demographic and lifestyle factors.

First, stroke was self-reported, which was prone to information error. Self-reports have proved to have poor sensitivity, but fairly good specificity and positive predictive values. Second, covariates were all collected in baseline and the changes had not been considered. Third, for the subtypes of stroke (such as ischemic stroke and hemorrhagic stroke) had not been distinguished in CHARLS, we could not analyze the association between HGS/D-HGS and incidence of stroke by subtypes. Finally, although we have adjusted for many potential confounders, we could not completely rule out the possibility of residual confounding by factors that had not been included in the analysis for sample size consideration, such as exercises, and unmeasured factors, such as dietary intake.

## CONCLUSIONS

HGS weakness was significantly associated with an increased risk of prevalence and incidence of stroke. The status of HGS weakness lasting for four years and HGS declined from normal at baseline to weakness after four years were also significantly associated with stroke incidence, even stronger. HGS could be used as a quick, low-cost screening tool by doctors or other healthcare professionals to identify high-risk patients who are prone to having stroke.

## MATERIALS AND METHODS

### Study populations

The China Health and Retirement Longitudinal Study (CHARLS), conducted by the National School for Development (China Center for Economic Research), is a large-scale, multistage, and nationally representative ongoing health survey of the Chinese population [30, 31]. Samples of households with members aged 45 and older were chosen, and a total of 17,708 individuals in 10,257 households, 450 villages nationwide, 150 counties, and districts from 28 provinces were selected in the baseline year (2011) [32]. All participants were required to sign informed consent. Ethics approval for data collection for CHARLS was obtained from the Biomedical Ethics Review Committee of Peking University (IRB00001052–11015).

**Figure 3:**
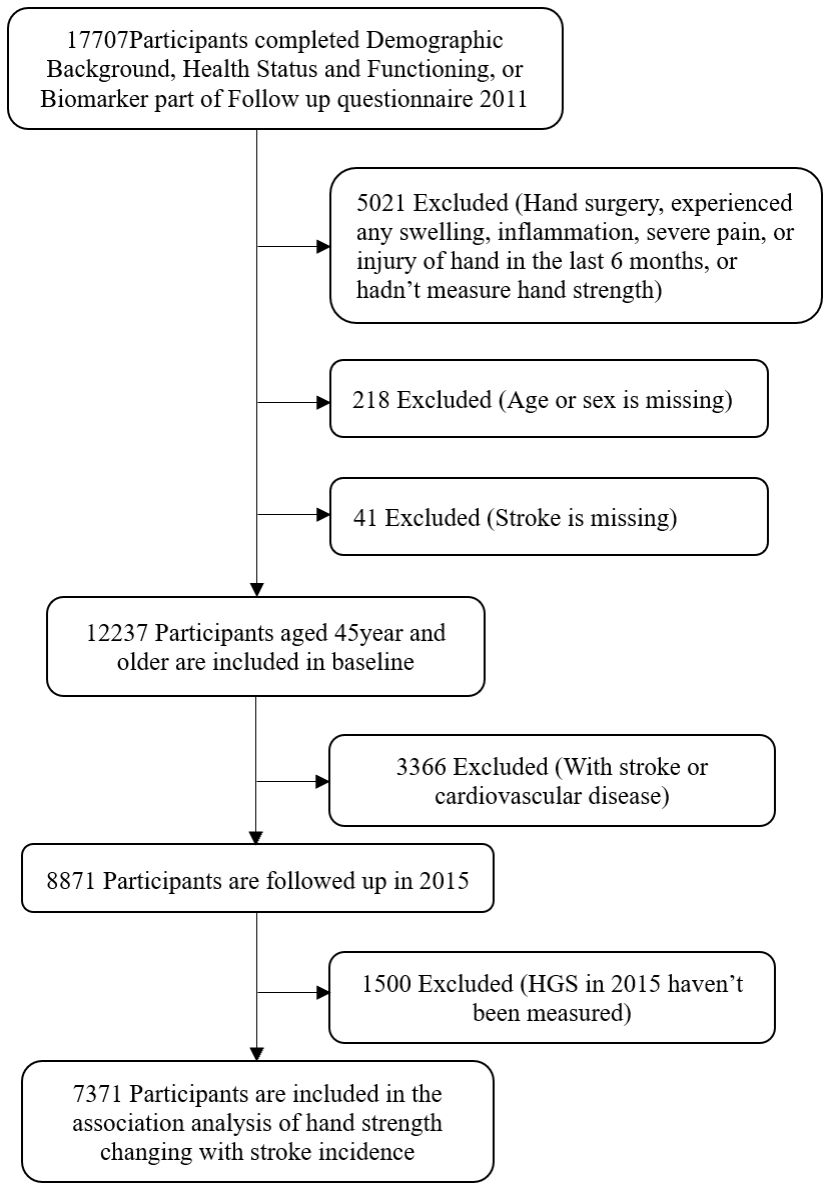
**Flowchart of participant selection.**

HGS and covariates from 12,237 participants aged 45 years and older were acquired in CHARLS 2011. The 8,871 participants who were free of a history of stroke and cardiovascular disease at baseline were followed up in 2015. Of that number, the 7371 participants whose HGS was measured in 2015 were compared against the 2011 baseline to determine the association of changes in HGS with stroke incidence. The flowchart of participant selection was showed in Figure 3.

### Data availability statement

All the data relevant for the manuscript are reported in tables. The raw data can be accessed from the corresponding author upon request.

### Outcome assessment

Stroke was self-reported and diagnosed with one question: “Have you ever been diagnosed with stroke by a doctor?” Researchers checked the medicine they were taking or the receipts in their clinical records for those who reported having a stroke.

### Exposure measurement

We measured HGS, and changes of HGS, from 2011 to 2015 (D-HGS) as exposures. HGS was measured in kilograms utilizing an electronic dynamometer. Electronic dynamometer has similar accuracy to Jamar dynamometer, with low price and more universal [33]. Before measurement, investigators demonstrated the procedure: stand, hold the dynamometer (measure force in kilograms) at a right angle and squeeze the handle for a few seconds. Each hand was measured twice; the mean value of the dominant hand was taken as HGS. If one or two hands had surgery or experienced any swelling, inflammation, severe pain, or injury in the last 6 months, it would not be measured. Participants whose dominant hand could not be measured were removed from the study. HGS in 2011 was grouped either into a normal group or weakness group by FNIH Sarcopenia cut-off points for muscle weakness: men had HGS <26.0 kg and women <16.0 kg. Men with HGS ≥26.0kg and women ≥16.0kg were grouped into normal HGS group [32]. HGS in 2011 was used in the association analysis of HGS and stroke prevalence [34]. According to the HGS status in 2011 and 2015, we calculated D-HGS and classified participants into four groups: normal/normal, weakness/normal, weakness/weakness and normal/weakness.

### Covariate assessment

Based on previous works on stroke, we included several relevant covariates: age, gender, marriage status, education, place of residence, smoking behavior, alcoholic intake, body mass index (BMI), hypertension, fasting plasma glucose, total cholesterol, and C-reactive protein (CRP), that were measured by standard methods or collected using standardized questionnaires during interviews.

Marital status was categorized as married (married and living as married), other marital status (divorced or widowed, never married, and separated). Education was categorized into five groups: below primary school, primary school, junior middle school, high school, and junior college and above. Place of residence was discriminated as village, city or town, and other places. Smoking behavior was categorized as not smoking and smoking. Alcoholic intake was classified into drink more than once a month, drink less than once a month, and never had a drink.

BMI was calculated with weight(kg)/height(m)^2^. Height was measured using stadiometer. Participants were asked to stand erect on the floor board of the stadiometer with back to the vertical backboard of the stadiometer, weight evenly distributed on both feet, and heels of the feet placed together with both heels touching the base of the vertical board. The equipment used in weight measurement was scale. Participants were asked to stand on the scale with shoes off and then read the weight measurement (kg). The blood metrics, such as fasting plasma glucose, total cholesterol, and CRP were collected by staff of the Chinese Center for Disease Control and Prevention (China CDC) in the China CDC and Youanmen Center for Clinical Laboratory of Capital Medical University. Fasting plasma glucose and total cholesterol were measured by enzymatic colorimetric test, CRP were measured by immunoturbidimetric assay. Blood pressure was measured using Omron HEM-7200 Monitor, batteries, and stopwatch. Systolic blood pressure (SBP) and diastolic blood pressure (DBP) was measured three times and mean values were taken. According to “Chinese guidelines for prevention and treatment of hypertension revised in 2018”, hypertension was diagnosed when SBP≥140mmHg or DBP≥90mmHg [35].

### Statistical analysis

Means, medians, and proportions of baseline characteristics were compared by using two independent *t* tests, Wilcoxon W test and χ^2^ test, respectively. The association of HGS and stroke prevalence was investigated by Logistic regression models based on the baseline information. The association of HGS and stroke incidence was analyzed by Cox proportional hazards regression models, where HGS and covariates were collected in baseline and stroke in 2015. Four models were conducted: Unadjusted Model; Model 1 considered age and gender, marriage status, education level and place of residence; Model 2 added smoking behavior and alcoholic intake based on Model 1; Model 3 added BMI, hypertension, glucose, total cholesterol, and C-reactive protein based on Model 2. We have checked model assumptions for all the analyses. Since there are few missing data covariates, no additional assumptions were made. All statistical analyses were conducted using IBM SPSS software package version 20 (Armonk, NY, IBM Corp.). Two-sided P values <0.05 was considered statistically significant.

## References

[1] Feigin VL, Lawes CM, Bennett DA, Barker-Collo SL, Parag V. Worldwide stroke incidence and early case fatality reported in 56 population-based studies: a systematic review. Lancet Neurol. 2009; 8:355–69. 10.1016/S1474-4422(09)70025-0 19233729

[2] Donnan GA, Fisher M, Macleod M, Davis SM. Stroke. Lancet. 2008; 371:1612–23. 10.1016/S0140-6736(08)60694-7 18468545

[3] Yang G, Wang Y, Zeng Y, Gao GF, Liang X, Zhou M, Wan X, Yu S, Jiang Y, Naghavi M, Vos T, Wang H, Lopez AD, Murray CJ. Rapid health transition in China, 1990-2010: findings from the global burden of disease study 2010. Lancet. 2013; 381:1987–2015. 10.1016/S0140-6736(13)61097-1 23746901 PMC7159289

[4] Global Burden of Disease Collaborative Network. Global Burden of Disease Study 2017 (GBD 2017) Results. 2018. http://ghdx.healthdata.org/gbd-results-tool.

[5] Go AS, Mozaffarian D, Roger VL, Benjamin EJ, Berry JD, Blaha MJ, Dai S, Ford ES, Fox CS, Franco S, Fullerton HJ, Gillespie C, Hailpern SM, et al, and American Heart Association Statistics Committee and Stroke Statistics Subcommittee. Executive summary: heart disease and stroke statistics—2014 update: a report from the American heart association. Circulation. 2014; 129:399–410. 10.1161/01.cir.0000442015.53336.12 24446411

[6] Hankey GJ. Ischaemic stroke—prevention is better than cure. J R Coll Physicians Edinb. 2010; 40:56–63. 10.4997/JRCPE.2010.111 21125042

[7] Xia X, Yue W, Chao B, Li M, Cao L, Wang L, Shen Y, Li X. Prevalence and risk factors of stroke in the elderly in northern China: data from the national stroke screening survey. J Neurol. 2019; 266:1449–58. 10.1007/s00415-019-09281-5 30989368 PMC6517347

[8] Ovbiagele B, Nguyen-Huynh MN. Stroke epidemiology: advancing our understanding of disease mechanism and therapy. Neurotherapeutics. 2011; 8:319–29. 10.1007/s13311-011-0053-1 21691873 PMC3250269

[9] Murray CJ, Barber RM, Foreman KJ, Abbasoglu Ozgoren A, Abd-Allah F, Abera SF, Aboyans V, Abraham JP, Abubakar I, Abu-Raddad LJ, Abu-Rmeileh NM, Achoki T, Ackerman IN, et al, and GBD 2013 DALYs and HALE Collaborators. Global, regional, and national disability-adjusted life years (DALYs) for 306 diseases and injuries and healthy life expectancy (HALE) for 188 countries, 1990-2013: quantifying the epidemiological transition. Lancet. 2015; 386:2145–91. 10.1016/S0140-6736(15)61340-X 26321261 PMC4673910

[10] Kamide N, Kamiya R, Nakazono T, Ando M. Reference values for hand grip strength in Japanese community-dwelling elderly: a meta-analysis. Environ Health Prev Med. 2015; 20:441–46. 10.1007/s12199-015-0485-z 26253392 PMC4626464

[11] Bohannon RW. Grip strength: an indispensable biomarker for older adults. Clin Interv Aging. 2019; 14:1681–91. 10.2147/CIA.S194543 31631989 PMC6778477

[12] McGrath RP, Kraemer WJ, Snih SA, Peterson MD. Handgrip strength and health in aging adults. Sports Med. 2018; 48:1993–2000. 10.1007/s40279-018-0952-y 29943230

[13] Sayer AA, Kirkwood TB. Grip strength and mortality: a biomarker of ageing? Lancet. 2015; 386:226–27. 10.1016/S0140-6736(14)62349-7 25982159

[14] Cooper R, Kuh D, Cooper C, Gale CR, Lawlor DA, Matthews F, Hardy R, and FALCon and HALCyon Study Teams. Objective measures of physical capability and subsequent health: a systematic review. Age Ageing. 2011; 40:14–23. 10.1093/ageing/afq117 20843964 PMC3000177

[15] Sayer AA, Robinson SM, Patel HP, Shavlakadze T, Cooper C, Grounds MD. New horizons in the pathogenesis, diagnosis and management of sarcopenia. Age Ageing. 2013; 42:145–50. 10.1093/ageing/afs191 23315797 PMC3575121

[16] Bertrand AM, Fournier K, Wick Brasey MG, Kaiser ML, Frischknecht R, Diserens K. Reliability of maximal grip strength measurements and grip strength recovery following a stroke. J Hand Ther. 2015; 28:356–62. 10.1016/j.jht.2015.04.004 26206167

[17] Park JG, Lee KW, Kim SB, Lee JH, Kim YH. Effect of decreased skeletal muscle index and hand grip strength on functional recovery in subacute ambulatory stroke patients. Ann Rehabil Med. 2019; 43:535–43. 10.5535/arm.2019.43.5.535 31693843 PMC6835132

[18] Sunderland A, Tinson D, Bradley L, Hewer RL. Arm function after stroke. An evaluation of grip strength as a measure of recovery and a prognostic indicator. J Neurol Neurosurg Psychiatry. 1989; 52:1267–72. 10.1136/jnnp.52.11.1267 2592969 PMC1031635

[19] Zhang W, Zhang T. Association between Grip Strength and Cognitive Function among Middle-aged and Older Adults with Stroke: Findings from the China Health and Retirement Longitudinal Study. Chin J Rehabil Theory Pract. 2019; 25:279–83. 10.3969/j.issn.1006-9771.2019.03.006

[20] Leong DP, Teo KK, Rangarajan S, Lopez-Jaramillo P, Avezum A Jr, Orlandini A, Seron P, Ahmed SH, Rosengren A, Kelishadi R, Rahman O, Swaminathan S, Iqbal R, et al, and Prospective Urban Rural Epidemiology (PURE) Study investigators. Prognostic value of grip strength: findings from the prospective urban rural epidemiology (PURE) study. Lancet. 2015; 386:266–73. 10.1016/S0140-6736(14)62000-6 25982160

[21] Park S, Park JY. Grip strength in post-stroke hemiplegia. J Phys Ther Sci. 2016; 28:677–79. 10.1589/jpts.28.677 27065562 PMC4793032

[22] Smith L, Yang L, Hamer M. Handgrip strength, inflammatory markers, and mortality. Scand J Med Sci Sports. 2019; 29:1190–96. 10.1111/sms.13433 30972827

[23] Liu Y, Wang J, Zhang L, Wang C, Wu J, Zhou Y, Gao X, Wang A, Wu S, Zhao X. Relationship between C-reactive protein and stroke: a large prospective community based study. PLoS One. 2014; 9:e107017. 10.1371/journal.pone.0107017 25191699 PMC4156395

[24] Kernan WN, Ovbiagele B, Black HR, Bravata DM, Chimowitz MI, Ezekowitz MD, Fang MC, Fisher M, Furie KL, Heck DV, Johnston SC, Kasner SE, Kittner SJ, et al, and American Heart Association Stroke Council, Council on Cardiovascular and Stroke Nursing, Council on Clinical Cardiology, and Council on Peripheral Vascular Disease. Guidelines for the prevention of stroke in patients with stroke and transient ischemic attack: a guideline for healthcare professionals from the American Heart Association/American Stroke Association. Stroke. 2014; 45:2160–36. 10.1161/STR.0000000000000024 24788967

[25] Lawman HG, Troiano RP, Perna FM, Wang CY, Fryar CD, Ogden CL. Associations of relative handgrip strength and cardiovascular disease biomarkers in U.S. Adults, 2011-2012. Am J Prev Med. 2016; 50:677–83. 10.1016/j.amepre.2015.10.022 26689977 PMC7337414

[26] Sarfo FS, Mobula LM, Plange-Rhule J, Ansong D, Ofori-Adjei D. Incident stroke among Ghanaians with hypertension and diabetes: a multicenter, prospective cohort study. J Neurol Sci. 2018; 395:17–24. 10.1016/j.jns.2018.09.018 30268724 PMC6227375

[27] Cooper R, Kuh D, Hardy R, and Mortality Review Group, and FALCon and HALCyon Study Teams. Objectively measured physical capability levels and mortality: systematic review and meta-analysis. BMJ. 2010; 341:c4467. 10.1136/bmj.c4467 20829298 PMC2938886

[28] Forrest KY, Williams AM, Leeds MJ, Robare JF, Bechard TJ. Patterns and correlates of grip strength in older Americans. Curr Aging Sci. 2018; 11:63–70. 10.2174/1874609810666171116164000 29150988

[29] Saunders DH, Sanderson M, Hayes S, Kilrane M, Greig CA, Brazzelli M, Mead GE. Physical fitness training for stroke patients. Cochrane Database Syst Rev. 2016; 3:CD003316. 10.1002/14651858.CD003316.pub6 27010219 PMC6464717

[30] Zhao Y, Hu Y, Smith JP, Strauss J, Yang G. Cohort profile: the China health and retirement longitudinal study (CHARLS). Int J Epidemiol. 2014; 43:61–68. 10.1093/ije/dys203 23243115 PMC3937970

[31] Yu T, Ma J, Jiang Y, Li J, Gen Y, Wen Y, Sun W. Assessing pain among Chinese elderly-Chinese health and retirement longitudinal study. Iran J Public Health. 2018; 47:553–60. 29900140 PMC5996333

[32] Celis-Morales CA, Welsh P, Lyall DM, Steell L, Petermann F, Anderson J, Iliodromiti S, Sillars A, Graham N, Mackay DF, Pell JP, Gill JM, Sattar N, Gray SR. Associations of grip strength with cardiovascular, respiratory, and cancer outcomes and all cause mortality: prospective cohort study of half a million UK biobank participants. BMJ. 2018; 361:k1651. 10.1136/bmj.k1651 29739772 PMC5939721

[33] Massy-Westropp N, Rankin W, Ahern M, Krishnan J, Hearn TC. Measuring grip strength in normal adults: reference ranges and a comparison of electronic and hydraulic instruments. J Hand Surg Am. 2004; 29:514–19. 10.1016/j.jhsa.2004.01.012 15140498

[34] Li RC, Yang M, Zhu Y, Bao T, Tan LL, Zuo Y, Hu XY, Tang HR. A Diagnostic Cut-off Point of Grip Reduction in Chinese Adults Based on Physical Examination. Journal of Chengdu Medical College. 2020; 15:332–35, 343.

[35] Revised Committee of Chinese guidelines for prevention and treatment of hypertension. Chinese guidelines for prevention and treatment of hypertension revised in 2018. Prevention and Treatment of Cardio-Cerebral-Vascular Disease. 2019; 19:1–44.

